# Odor Identification in Rats: Behavioral and Electrophysiological Evidence of Learned Olfactory-Auditory Associations

**DOI:** 10.1523/ENEURO.0102-19.2019

**Published:** 2019-08-08

**Authors:** Jonas K. Olofsson, Guangyu Zhou, Brett S. East, Christina Zelano, Donald A. Wilson

**Affiliations:** 1 Emotional Brain Institute, Nathan S. Kline Institute, Orangeburg, NY 10962; 2 Department of Child and Adolescent Psychiatry, New York University School of Medicine, New York, NY 10016; 3 Department of Psychology, Stockholm University, Stockholm SE-10691, Sweden; 4 Department of Neurology, Northwestern University Feinberg School of Medicine, Chicago, IL 60611; 5 Department of Neuroscience and Physiology, New York University School of Medicine, New York, NY 10016

**Keywords:** auditory perception, electrophysiology, learning, olfaction, sensation

## Abstract

The ability to recognize and identify a smell is highly dependent on multisensory context and expectation, for example, hearing the name of the odor source. Here, we develop a novel auditory-odor association task in rats, wherein the animal learns that a specific auditory tone, when associated with a specific odor, predicts reward (Go signal), whereas the same tone associated with a different odor, or vice versa, is not (No-Go signal). The tone occurs prior to the onset of the odor, allowing physiological analyses of sensory-evoked local field potential (LFP) activity to each stimulus in primary auditory cortex and anterior piriform cortex (aPCX). In trained animals that have acquired the task, both auditory and subsequent olfactory cues activate β band oscillations in both the auditory cortex and PCX, suggesting multisensory integration. Naive animals show no such multisensory responses, suggesting the response is learned. In addition to the learned multisensory evoked responses, functional connectivity between auditory cortex and PCX, as assessed with spectral coherence and phase lag index (PLI), is enhanced. Importantly, both the multi-sensory evoked responses and the functional connectivity are context-dependent. In trained animals, the same auditory stimuli presented in the home cage evoke no responses in auditory cortex or PCX, and functional connectivity between the sensory cortices is reduced. Together, the results demonstrate how learning and context shape the expression of multisensory cortical processing. Given that odor identification impairment is associated with preclinical dementia in humans, the mechanisms suggested here may help develop experimental models to assess effects of neuropathology on behavior.

## Significance Statement

An important feature in mammalian olfaction is the multisensory support provided by “higher” senses, such as hearing and vision. In humans, such multisensory context and expectation, for example, hearing the name of the odor source, facilitates the identification of a smell. An impaired ability to identify odors is a sensitive predictor of cognitive decline and neurodegenerative dementia. We found that rats trained on a tone-odor association task, but not untrained rats, showed elevated electrophysiological responses in both auditory and olfactory cortices, as well as increased functional connectivity between these regions, during task engagement. These results provide evidence of a multisensory integration process that might provide clues to how neuropathology affects the brain.

## Introduction

Multisensory processing allows activity in one sensory pathway to modulate activity in another, in turn shaping perception. For example, in the McGurk effect, discordant visual cues influence auditory perception to the point where a subject hears a syllable consistent with the visual mouth movements of the speaker instead of the actual auditory syllable (McGurk and MacDonald, 1976). The convergence of sensory processing streams that enables multisensory perception has been described in increasingly earlier stages of “unimodal” sensory pathways, including classic primary sensory areas (Calvert et al., 1999; Foxe et al., 2000). Importantly, more recent evidence suggests that multisensory integration can be context-dependent, and not a stable result of fixed anatomic convergence (Schroeder et al., 2001; Choi et al., 2018).

The vast majority of work on multisensory processing has occurred in classic thalamocortical sensory systems such as vision and audition. However, multisensory integration also occurs in the olfactory system (Gottfried and Dolan, 2003; Wesson and Wilson, 2010; Cohen et al., 2011; Mandairon et al., 2014; Karunanayaka et al., 2015; Boisselier et al., 2018), an evolutionarily older system with no thalamic relay between the periphery and the olfactory cortex, which itself is a three-layered archicortex (Neville and Haberly, 2004; Wilson and Sullivan, 2011; Bekkers and Suzuki, 2013). For example, visual cues can influence olfactory perception, e.g., red liquids with strawberry scent smell more intense than clear liquids (Zellner and Kautz, 1990; Morrot et al., 2001; Demattè et al., 2009), as can gustatory, somatosensory and auditory cues (Cain, 1979; Dalton et al., 2000; Stevenson and Mahmut, 2011; Stevenson et al., 2012). Much of the multisensory influences on olfactory perception are related to semantic associations of the odor (i.e., identifiability or familiarity; de Araujo et al., 2005; Stevenson et al., 2012), nonetheless, multisensory stimulation is associated with modulation of odor-evoked activity in central olfactory and limbic regions in humans (Gottfried and Dolan, 2003; de Araujo et al., 2005; Karunanayaka et al., 2015; Zhou et al., 2019) and animal models (Kadohisa et al., 2005; Wesson and Wilson, 2010; Mandairon et al., 2014; Maier et al., 2015; Veyrac et al., 2015).

A vulnerability in links between the olfactory system and other multisensory and language related regions has been hypothesized to underlie the sensitivity of odor identification to a variety of disorders including mild cognitive impairment, Alzheimer’s disease and primary progressive aphasia (Devanand et al., 2008; Olofsson and Gottfried, 2015; Olofsson et al., 2009, 2013; MacDonald et al., 2018). However, recent evidence suggests that an auditory word cue that primes a matching odor may generate a strong functional coupling between primary olfactory cortex and higher order auditory cortex in humans (Zhou et al., 2019). Specifically, subjects prepared for intracranial electroencephalography were presented with an auditory word cue of an odor name followed by a matching or non-matching odor. Following the auditory word, but before delivery of the odor, there was strong phase locking of local field potentials (LFPs) between auditory and olfactory cortices (Zhou et al., 2019).

As auditory or visual stimuli may cause effective predictive projections to olfactory cortex, facilitating cross-sensory integration and odor identification, and as this ability appears especially vulnerable to effects of common neuropathological disorders, research on the physiologic mechanisms underlying this task is warranted. We developed a novel auditory-odor association task in rats, wherein the animal must learn that a specific auditory tone, when associated with a specific odor, predicts reward (Go signal), whereas the same tone associated with a different odor, or vice versa, does not (No-Go signal). This design may be characterized as a simple odor identification task, as one odor needs to be matched to the correct non-olfactory cue. Because of the slight lag between tones and odors, this design also allowed analyses of separate auditory and olfactory driven activity within the olfactory and auditory cortices. We explored the effects of learning on multisensory-evoked activity within auditory and olfactory cortex, as well as the effects of context on this activity. Together, the results demonstrate the importance of both learning and context in expression of multisensory cortical processing in olfaction.

## Materials and Methods

### Subjects

Male Long Evans hooded rats (*n* = 15; 200–450 g) from Envigo Lab Animals were used as subjects. Animals were single housed with enrichment and had *ad libitum* access to food. Access to water was restricted during the training session and for 30 min 2–4 h after training while in the home cage. Body weight and health status were monitored daily and no rat declined under 85% of its initial weight over the course of training. Lights were on from 6 A.M. to 6 P.M. and all testing occurred in the light portion of the day. All handling, housing and experimental procedures were approved by, and performed in accordance with the Institutional Animal Care and Use Committee guidelines as well as National Institutes of Health guidelines for the proper treatment of animals. The animals in this experiment are referred to as odor-tone (OT)1, OT2, etc., when data from individual animals are presented.

### Behavioral assay

Go/No-Go training occurred in a black Plexiglas operant chamber (27 × 27 × 33 cm; width × length × height) with an infrared (IR) beam-monitored nose port on the back wall and an IR beam-controlled water port on a side wall. Breaking the beam in the nose port initiated a trial with a 90-dB sine wave tone (0.1 or 1 kHz) lasting 500 ms. Immediately after the end of the tone, an odor (peppermint or vanilla, McCormick) was delivered through the same nose port, and was maintained until the animal left the port. If the rat left the port <500 ms following the tone onset, the trial was considered aborted. The animals were required to learn that a specific combination of tone and odor was the stimulus that signaled an available water reward (100 µl; Go signal), if the animal broke the IR beam in the water port within 3 s. Other combinations of tone and odor were not rewarded (No-Go signals). Following initial shaping, the probability of a Go trial was 60%. During training sessions, all animals were held in the testing room, and thus had passive exposure to the sound of the tones generated by other rats. During initial shaping, in the first few days, the minimal nose-poke duration was 200 ms, Go trial combinations occurred at 80% of trials, and all nose-pokes led to water reward. As the rats learned the task, these initial task settings were changed gradually during the first 10 d, according to a standardized protocol, such that the final settings were 500-ms poke, 60% Go trials, and no reward on No-Go trials.

### Electrophysiology

To examine cortical processing of multisensory signals, a subset of animals (well-trained, *n* = 8; naive *n* = 6) were used for LFP recording from the auditory cortex (A1) and anterior piriform cortex (aPCX). In addition, four animals were not able to learn the multisensory task after surgery, data from these animals were not considered. Rats were implanted with electrodes (stainless steel, 127 µm) directed toward A1 (3.5 mm posterior to bregma, 6.5 mm lateral, 5.0 mm ventral) and toward aPCX (1.0 mm anterior to bregma, 4.5 mm lateral, 8.0 mm ventral) to record LFPs from these regions during behavioral training. The electrodes were attached to a telemetry devise implanted under the dorsal skin (F20-EET, DSI, Inc). Analog signals were transmitted to a receiver placed under the operant chamber or under the homecage, digitized at 5 kHz, and collected and analyzed using Spike2 software (Cambridge Electronic Design). Given that the tone and odor stimuli did not temporally overlap, LFP responses to both could be extracted independently. LFP activity during the tone (500-ms period) and odor (250-ms period) stimuli was analyzed with fast Fourier transform (FFT; 4.9-Hz bins), as well as activity during periods when the animal was disengaged from the task (i.e., not self-initiating trials for >30 s) which served as baseline. In addition to stimulus evoked activity during the Go/No-Go task, LFPs were also recorded when the animals were in their homecage. For these recordings, the homecage was placed next to the operant chamber while another rat was in training and generating trials. In this condition, the tones were audible in the homecage at the same intensity (90 dB) as in the operant chamber. This allowed analyses of tone evoked activity in animals while in the context of their homecage. Baseline LFP activity in the homecage during periods of no tones was also quantified. Given the design of the operant chamber and the brief duration of the stimulus, odors are presumed to not penetrate the homecage. For analysis of evoked activity in both the operant chamber and in the home cage, LFP activity during stimulation was expressed as a ratio of baseline activity.

In addition to stimulus evoked activity, spectral coherence of the power of LFP oscillations between A1 and aPCX (9.7-Hz bins) was examined using the cohere script in Spike2. A1–aPCX coherence was measured in three different states. (1) During active sampling. Data were obtained from 1 s of data starting at trial onset for each of the first 100 trials, regardless of trial type, within a session. (2) When the animal was in the operant chamber but disengaged from the task (>30-s period). For this analysis, coherence was determined across the full period of time (not trial dependent). (3) When the animal was in their home cage while another rat was in training and generating trials (> 30 s of tone-free activity, again not trial dependent).

An additional measure of functional connectivity was applied to more closely examine trial-related connectivity. To examine tone-evoked changes in phase locking between A1 and aPCX during trials, we calculated the phase lag index (PLI; Stam et al., 2007) across trials. PLI is a measure of phase synchrony with reduced bias from common sources and volume conduction (Stam et al., 2007). In brief, LFPs were bandpass filtered at log-spaced frequencies ranging from 1 to 30 Hz with a fixed bandwidth of 2 Hz. Instantaneous phase time series were extracted for A1 and aPCX using the Hilbert transformation. The resulting between-region phase difference time series were segmented into epochs from 0.5 s before 2 s following tone onset. Then, PLI was calculated at each time-frequency point and baseline corrected by subtracting the baseline (0.5 s before tone onset) average. Finally, the statistical significance of the PLI change was evaluated using a permutation method. For each permutation, the trial indices for one channel (A1/aPCX) were shuffled and the permuted PLI was calculated. After repeating this procedure 5000 times, we obtained a distribution of permuted PLI values at each time-frequency point. The mean and standard deviation of this distribution was obtained by normal distribution fitting (MATLAB’s *normfit*). A *z* score of the real PLI was then calculated by subtracting the mean of the distribution which was further divided by the standard deviation. Multiple comparison corrections were controlled using a cluster-based method. The *p* value threshold for initial clustering was set at 0.001 (two-sided, corresponding to a *z* score of 3.29). The maximal cluster size (sum of absolute *z* score) was retrieved for each permutation, resulting in a permuted cluster-size distribution. Corrected *p* values of each cluster from the real PLI *z* score map were calculated as the proportion of permuted clusters that were larger than the real one.

We also compared the PLI change between Go and No-Go trials, and between training and homecage sessions using a cluster-based correction method. In each permutation, trials from both conditions were pooled together and each trial was assigned to one of the conditions randomly. Then, permuted PLI (baseline corrected) was calculated for each condition and its between-condition difference was obtained. The real between-condition difference was tested against the permuted difference in a similar was described above using the same initial *p* value threshold (5000 permutations). To account for the difference in number of trials between different conditions, we estimated the PLI for the condition with a greater number of trials using a bootstrapping method. In each bootstrap, the PLI was calculated for a subset of trials (same number of the other condition) was randomly extracted from all trials. The above procedure was performed 200 times, and the average of all bootstraps was used as the final result.

## Results

**Figure 1.**
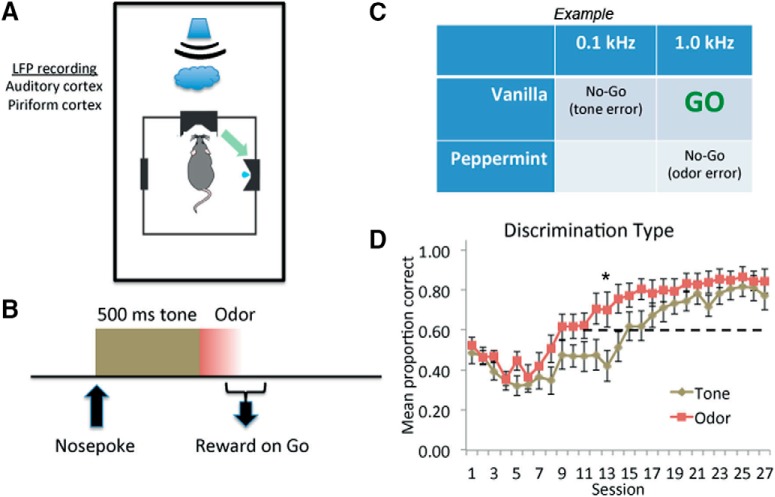
Experimental design and acquisition performance. ***A***, Rats initiated trials by nose poking a port which caused (***B***) onset of one of two 500-ms, 90-dB tones. At tone offset, one of two odors were presented through the port. ***C***, Odors included vanilla and peppermint and the frequency of the tones were 0.1 and 1 kHz. Different tone-odor associations served as the Go stimulus in different animals, although in all cases successful identification of the Go stimulus required matching the correct tone with the correct odor. ***D***, Acquisition of correct performance, divided by error type (mean ± SEM). Rats relatively quickly learned to reduce odor-based errors, but took longer to reduce tone-based errors. The horizontal dashed line represents chance performance. Initial decrease in performance is due to the fact that the task became progressively more difficult during the first 10 d of training (see Materials and Methods). The asterisk signifies a significant difference between Tone and Odor (*p* < 0.05).

The experimental design and behavioral results are shown in Figure 1. Rats learned to associate an auditory tone of a specific frequency to a specific odor to receive a water reward (Go trials). As shown in Figure 1*D*, rats attained a mean of 80% correct, although performance acquisition varied based on error type. As shown in Figure 1*C*, there could be two error types on Go trials. If, for example, the Go stimulus was the association of the higher frequency and vanilla, then a Go response to the combination of high frequency and peppermint would be an odor-based error (correct frequency but wrong odor). If, in contrast, a Go response occurred to the lower frequency tone and vanilla, this corresponds to a tone-based error (correct odor but wrong frequency). In animals performing above chance on the task, the mean sampling duration was 688 ± 20 ms (mean ± SEM) on Go trials and 701 ± 19 ms on No-Go trials. Trial type had no significant effect on sampling duration (Go trials, No-Go based on tone, No-Go based on odor, *F*_(3,18)_ = 1.03, *p* = 0.37). Thus, rats (*n* = 10) sampled the tone for the full 500 ms and the odor for nearly 200 ms. While rats ultimately had similar tone- and odor-based performance by the end of training (Fig. 1*D*), they learned odor-based correct responses significantly faster over the course of training than tone-based responses as shown by the significant interaction in a stimulus × trial, repeated measures ANOVA (2 × 27 repeated measures ANOVA: stimulus × trial interaction, *F*_(26,468)_ = 1.68, *p* = 0.02; main effect of trial, *F*_(1,26)_ = 37.24, *p* < 0.0001; main effect of stimulus, *F*_(1,18)_ = 2.69, *p* = 0.12).

**Figure 2.**
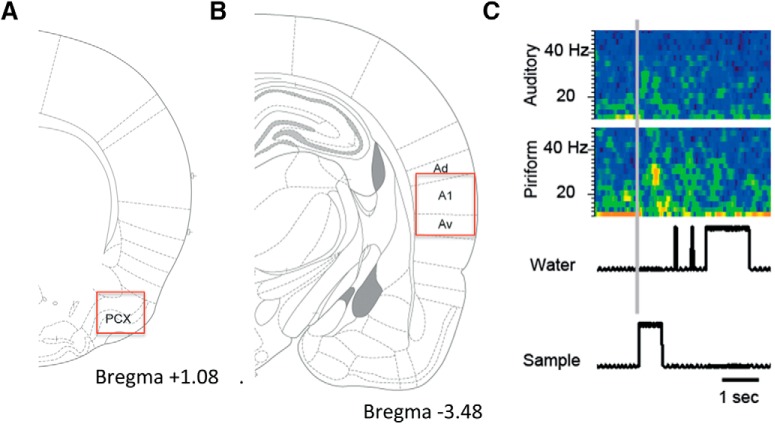
***A***, Location of aPCX electrode. ***B***, Location of A1 electrode. ***C***, Representative pseudocolor spectrogram (blue = low power, yellow = high power) of stimulus evoked oscillatory responses in A1 and aPCX during trial initiation in a well-trained rat. IR beam activity for the odor sampling port (sample) and water reward port (water) are shown at bottom aligned with the simultaneously recorded spectrogram.

To examine the effects of learning and context on oscillations in auditory and olfactory cortices, a subset of animals (*n* = 6) was implanted with electrodes before training onset, while the rest were implanted after they had attained criterion behavioral performance (>70% correct; Fig. 2). Due to technical limitations, no animals were recorded throughout the entire five- to six-week training session. LFP activity during the tone sampling period and the odor sampling period were expressed as a ratio of baseline and separately analyzed in both A1 and aPCX. In addition to recording during the training session, responses to tones were also recorded while the animals were in their homecages. The homecages were placed next to the operant chamber during a training session for a different rat. The sound intensity was confirmed to be the same in the operant chamber and the homecage.

**Figure 3.**
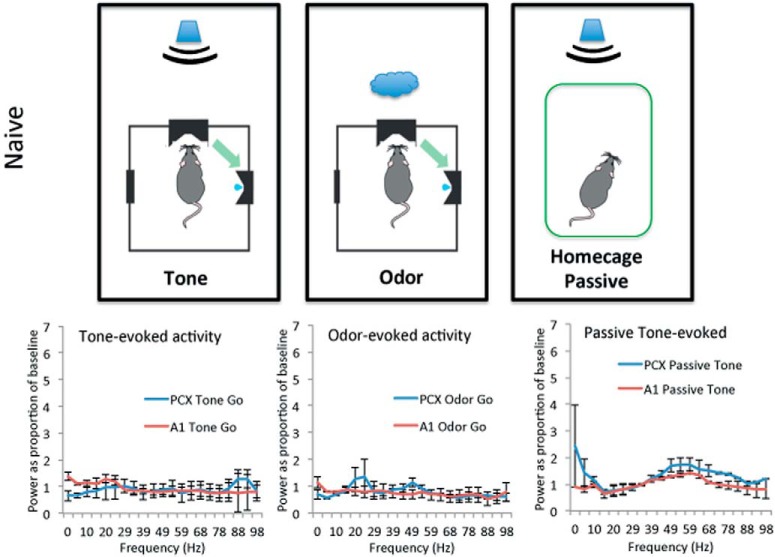
Stimulus evoked LFP activity in naive rats (during the initial two to three training sessions). LFP activity was assessed while the animals were actively initiating trials. Activity was quantified (1) during the 500 ms of tone presentation (tone-evoked); (2) for 250 ms starting at tone offset and odor onset (odor-evoked); and (3) during 500 ms of tone when the animals were in their home cage (passive tone-evoked). FFT analyses in each region to each stimulus were normalized as a ratio to baseline (disengagement from the task for at least 30 s), data displayed as mean ± SEM.

**Figure 4.**
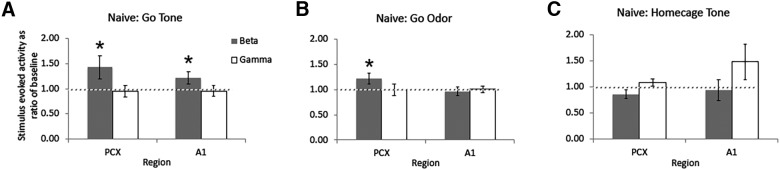
Quantification (mean ± 1 SEM) of stimulus-evoked β and γ band LFP power in naive rats. ***A***, Responses to Go tone. ***B***, Responses to Go odor. Activity was quantified in the same way as in Figure 3. No significant evoked response was observed to either tone or odor during the task, although tone was associated with a significant γ band response in aPCX. ***C***, Responses to tone while rats were in their homecage. Asterisk signifies significant difference from 1, *p* < 0.05 (PCX tone-evoked β, *p* = 0.044, A1 tone-evoked β, *p* = 0.047, PCX odor-evoked β, *p* = 0.032). The γ response in A1 was not significantly different from 1.0.

As shown in Figures 3, 4, in naive animals (*n* = 6) during initial two to three training sessions when performing at chance levels, small stimulus-evoked β band (15–35 Hz) oscillatory responses were observed in aPCX and A1. Figure 3 displays the full FFT spectrum, while Figure 4 shows the same data collapsed across the β and γ frequency bands for statistical analyses. β Band oscillations were significantly enhanced from baseline in both PCX and A1 in response to the tone, and in PCX in response to the odor (Fig. 3; one-sample *t* tests vs a mean of 1, df = 5, *p*s < 0.05). γ Band (35–90 Hz) oscillations remained near baseline levels (Fig. 4).

**Figure 5.**
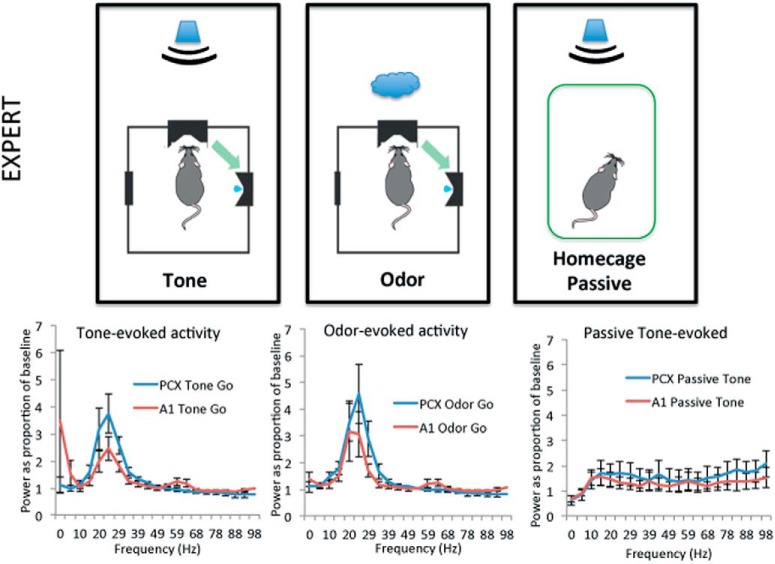
Stimulus evoked LFP activity in expert rats (mean ± 1 SEM). Note the robust β band activation in both aPCX and A1 in response to both the tone and odor while the animal was performing the task. This β band response to the tone was reduced while the animal was in their home cage. No evoked γ band activity was observed during training in either A1 or aPCX. FFTs show power as a percentage of baseline in 10-Hz bins starting at the frequency displayed.

**Figure 6.**
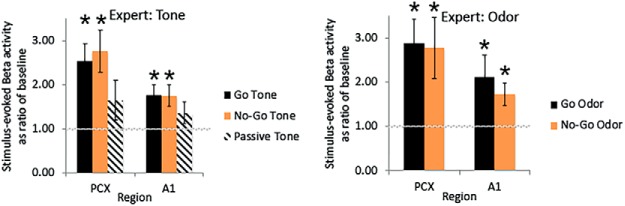
Quantification (mean ± 1 SEM) of stimulus-evoked β band LFP power to tone and odor in A1 and aPCX of Expert rats during the task and in the homecage. Both Go and No-Go tones and odors evoked significant β band activity in the aPCX and A1, suggesting multisensory activation of these cortices. Presentation of the same tone while the animals were in their homecage, however, produced no significant β band activity. Asterisks signify significant differences from 1, *p* < 0.05.

In contrast to these very weak sensory evoked responses in naive rats, in animals trained to criterion (expert, *n* = 8, with one animal having a failed aPCX electrode), robust multi-sensory (tones and odors) β band oscillations were evoked in both aPCX and A1 (Fig. 5). In contrast, responses to tones presented while the animal was in their homecage were greatly reduced in both structures compared to during active training. No γ band response above baseline was detected, thus statistical analyses were focused on β band activity. As shown in Figure 6, both aPCX and A1 displayed significant evoked β band activity in response to both tones and odors (significantly >1, one-sample *t* tests, all *p*s < 0.05). This was true for both tones that were a component of the Go signal and tones that were a component of the No-Go signal. The same tones presented while the animals were in their homecage, however, evoked no significant β band activity in either aPCX or A1 (one-sample *t* tests, *p* > 0.05). Similar to tones, both aPCX and A1 showed significant β band activity to Go and to No-Go associated odors (Fig. 6). It should be noted that the animals were not asleep during the homecage stimulation, as assessed with the LFP (data not shown).

**Figure 7.**
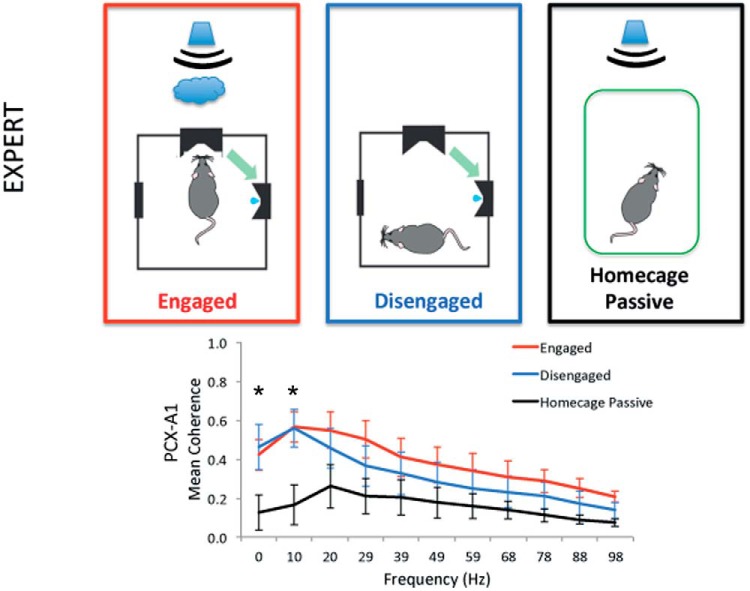
Spectral coherence between A1 and aPCX is state-dependent in well trained rats (mean ± 1 SEM). Graph shows coherence in 10-Hz bins starting at the frequency displayed. Coherence is enhanced while the animals are actively engaged in the task and generating trials, and while the animals are in the operant chamber but not actively engaged in the task for at least 30 s compared to when the animals are in their homecage while another animal is in the operant chamber and generating trials. Asterisks signify significant differences from the homecage condition, *p* < 0.05.

We next sought to determine if there was elevated functional connectivity between aPCX and A1 areas to indicate task- or context-dependent auditory-olfactory cortical communication. We compared the state-dependent multi-sensory activity in aPCX and A1 during training, relative to homecage activity (*n* = 7). aPCX–A1 LFP coherence was determined for three conditions: (1) active sampling; (2) disengagement from the task but while still in the operant chamber (defined as periods with no task activity of >30 s, animals were mostly grooming or exploring during this time); and (3) in the homecage while being exposed to tones generated during training by another rat. As shown in Figure 7, while rats were in the operant chamber, either actively engaged in the task or not engaged, aPCX–A1 coherence was significantly enhanced compared to being in the homecage (state × frequency repeated measures ANOVA, main effect of state, *F*_(2,132)_ = 23.37, *p* < 0.0001; main effect of frequency, *F*_(10,132)_ = 3.65, *p* = 0.0007; *post hoc* Tukey tests revealed a significant difference between both engaged and disengaged rats from homecage rats at the 0- to 10- and 10- to 20-Hz frequency bins).

**Figure 8.**
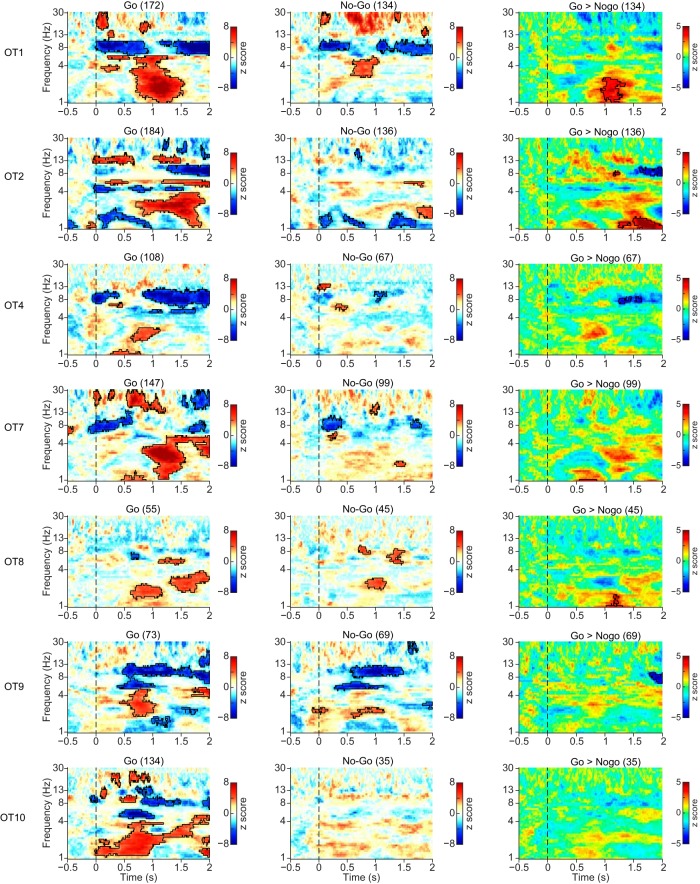
PLI analysis for seven individual rats for Go and No-Go trials, and the difference between trials. The PLI was baseline corrected ([−0.5, 0] s before tone onset). The significance of the increase was tested using a cluster-based permutation test. The threshold for initial clustering was *p* = 0.001. Outline regions indicate statistically significant clusters (*p* < 0.05). PLI increase difference between Go and No-Go trials. Most animals showed a significant increase in PLI beginning during the sampling period (sampling occurred 0–0.7 s) which extended into the reward period (1–2 s after trial onset). As shown in the right column, there was no consistent difference between Go and No-Go trials.

In addition to LFP coherence, we examined PLI between the LFPs recorded in A1 and aPCX in each rat. As can be seen in Figure 8, the PLI index (baseline corrected) between A1 and aPCX during Go trials was significantly elevated in a low frequency band (<5 Hz) during trials. In most animals, this enhanced PLI began near tone offset/odor onset (0.5 s) and extended through water reward (1–1.5 s after trial onset). In most (5/7) animals this same pattern was observed during No-Go trials. However, a direct comparison between Go and No-Go trials (Fig. 8, right column) showed rats OT1, OT2, and OT8 had significantly higher PLI during Go trials, primarily after the sampling period which may be related to reward consumption.

**Figure 9.**
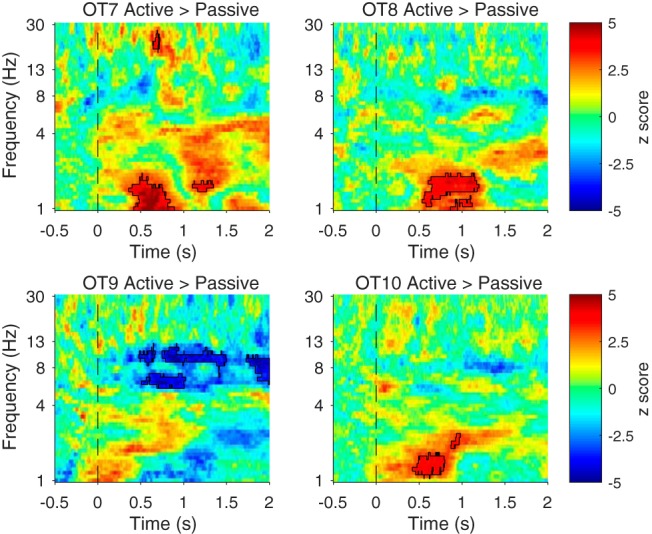
Tone induced stronger PLI increase while the animals were exposed to tones in the training session compared to the homecage. Data from animals tested in both conditions are shown as a difference between active and homecage conditions. The significance of the increase was tested using a cluster-based permutation test. The threshold for initial clustering was *p* = 0.001. Outlined regions indicate statistically significant clusters (*p* < 0.05).

We also analyzed A1–aPCX PLI in response to tones presented while the animals were in their homecage (*n* = 4). Again, similar to our LFP coherence analyses, the tone-evoked A1–aPCX connectivity increase that occurred during active training was significantly reduced when the animals were stimulated in their homecage (Fig. 9). Note, however, that while a consistent pattern is observed across OT7, OT8, and OT10, OT9 does not show any reduction.

## Discussion

Here, we describe a novel auditory-olfactory associative learning task that requires rats to learn to associate specific sounds with specific odors. The rats acquire this task relatively well, although show an initial bias toward odor cues. Within this task, rats demonstrate learning- and context-dependent multisensory processing in aPCX and A1. In well-trained rats, A1 displayed a robust β band oscillation in response to both learned tones and learned odors, as did the aPCX. These multisensory evoked responses were not observed in untrained rats. In addition to the multisensory evoked responses, functional coupling between aPCX and A1 was enhanced, as assessed with LFP coherence and phase locking. Importantly, both the evoked responses and the functional connectivity changes were only expressed while the rats were in the operant chamber, and not expressed when in their homecage. These results contribute to our understanding of multisensory processing effects in olfaction. Recognizing and identifying odors relies on effectively using predictive cues from other senses (e.g., when being asked to smell milk to check whether it is spoiled, we predict what the milk will smell like before odor sampling; Zelano et al., 2011). Integration of matching non-olfactory and olfactory cues is assessed in common clinical olfactory assessments (Doty et al., 1984; Hummel et al., 2007), and thus understanding the neural processing underlying such behavioral performance is critical.

In expert animals, both tone and odor stimuli evoked β oscillations in both A1 and aPCX. By definition, these appear to be multisensory evoked responses in classically unimodal cortices. In fact, links between the auditory and olfactory cortex in rodents have been well described (Budinger et al 2006; Budinger and Scheich 2009; Wesson and Wilson 2011). Furthermore, β band oscillations are typically associated with inter-regional communication (Tallon-Baudry et al., 2001; Engel and Fries, 2010) including within the olfactory system (Kay, 2014), and are sensitive to learning, typically increasing with behavioral improvement (Martin et al., 2004, 2007; Kay, 2014). However alternative mechanisms of multi-sensory evoked responses in A1 and aPCX are possible. For example, responses need not be directly driven by sensory afferents to the cortex itself, but rather could reflect changes in activity due to very indirect or even neuromodulatory circuits. For example, the temporal structure of PCX unit activity is shaped by locus coeruleus activation, causing unit firing to be more strongly in phase with respiration (Bouret and Sara, 2002). Thus, activation of the locus coeruleus, for example, by an alerting stimulus like foot shock, could evoke a change in PCX activity that was not truly a somatosensory evoked response, but rather a more general response to a change in neuromodulatory tone.

A role for involvement of neuromodulatory inputs in shaping the observed multisensory responses is further supported by the fact that they were context-dependent. The same auditory stimulus that evoked robust β band oscillations in A1 and aPCX while the animal was engaged in the task evoked no such activity when the animal was in their homecage. The change in context could modify attention or alertness, which can directly modify sensory-evoked responses in the olfactory (Carlson et al., 2018) and other sensory systems (Fritz et al., 2007; Dykstra et al., 2017; Moore and Zirnsak, 2017). Thus, it is difficult to distinguish between changes in context or changes in state as the driver of these effects. Further work is necessary to explore the underlying mechanisms of these learned and context-dependent multisensory responses.

In addition to the learned multisensory-evoked responses, functional connectivity between A1 and aPCX was also enhanced in a learning- and context-dependent manner. This effect was apparent using either LFP coherence over long (100 s) periods of time while the animal engaged in the task, or in trial-related phase-locking analyses (PLI). Given that the learned change in A1–aPCX functional connectivity was context-dependent suggests that stable synaptic plasticity, such as long-term potentiation, is not a primary mechanism. Rather, the data suggest a flexibility in multisensory processing, where under specific conditions sensory systems are more likely to function coherently, while a change in conditions allows them isolate from each other. Similar context-dependent expression of learned changes in network connectivity have been previously reported within the olfactory cortex itself (Cohen et al., 2015; Cohen and Wilson, 2017).

While the changes in aPCX and A1 LFP sensory-evoked power were in the β frequency band, the changes in functional connectivity were in lower frequencies (≤10 Hz). This may suggest that the sensory-evoked β band oscillations in aPCX and A1 are not imposed on the two structures by a third β band driving region, but rather locally generated. However, enhanced functional connectivity at low frequencies could contribute to local high frequency activity via cross-frequency coupling in the two regions, although this requires further investigation (Hyafil et al., 2015). The mechanisms of the low frequency PLI coupling between A1 and aPCX during sampling/reward are not clear. Our LFP coherence measures peaked in a similar low frequency range but were present both while the animals were actively engaged in the task and when they specifically were not, and instead were generally grooming or exploring. This would argue that active sniffing (6–9 Hz; Kepecs et al., 2007; Wesson et al., 2008) may not be not a major generator of A1–PCX connectivity here, although we did not monitor respiration directly. Furthermore, in humans performing an auditory-word-cued odor-matching task, high auditory-olfactory cortex coupling during the task was not respiration-dependent (Zhou et al., 2019). However, respiration has been shown to be important for linking activity within broad networks beyond the olfactory system in both humans (Zelano et al., 2016) and rats (Yanovsky et al., 2014; Lockmann et al., 2016; Moberly et al., 2018; Tort et al., 2018), and this respiration entrainment is important for odor memory and cognition (Zelano et al., 2016; Arshamian et al., 2018; Nakamura et al., 2018).

Finally, while odor detection and discrimination can be disrupted in a variety of disorders, odor identification, the linking of an odor sensation with a symbolic label, is generally most vulnerable to pathology associated with dementia (Murphy, 1999; Devanand et al., 2000) even in early and pre-clinical stages (Calhoun-Haney and Murphy, 2005; Olofsson et al., 2016; East et al., 2018). Similarly, animal models of dementia and dementia risk factors often show no deficits in odor discrimination (Vloeberghs et al., 2008; Phillips et al., 2011; Xu et al., 2014), despite impaired odor memory (Wesson et al., 2010) and olfactory system electrophysiological dysfunction (Wesson et al., 2011; East et al., 2018). More complex animal model tasks, such as the auditory-olfactory associative learning task characterized here, may more closely model multisensory links between olfaction and language that are so vulnerable in humans.

## Synthesis

Reviewing Editor: Julie Bakker, University of Liege Decisions are customarily a result of the Reviewing Editor and the peer reviewers coming together and discussing their recommendations until a consensus is reached. When revisions are invited, a fact-based synthesis statement explaining their decision and outlining what is needed to prepare a revision will be listed below. The following reviewer(s) agreed to reveal their identity: Michael Baum.

Both reviewers found your work potentially interesting and an advance to the field. However, there are some concerns regarding the description of the methods and presentation of the results. Please pay in particularly attention to the comments of reviewer 1 when revising your paper. Finally, please explain why the number of rats used is varying between the experiments.

Reviewer 1:

This work addresses the importance of context and task engagement in a multisensory integration process acquired through learning.

To my opinion, this study could provide a valuable advance in the field. It confirms learning-induced changes in electrophysiological activity of each involved sensory areas and addresses for the first time the impact of the experimental context and subject engagement in the task on functional connectivity between these two cortices in response to the same stimulus.

However, in the present way, the manuscript suffers from a lot of imprecisions and need to be thoroughly improved to be really convincing.

Main points:

Methods:

This section is not precise enough and the part on signal processing need to be reorganized in a more rigorous way.

For example, it is not clear how baseline LFP activity was determined both in the experimental cage and in the home cage. This point is critical as everything is then corrected by this baseline. Please provide a clear description of how baseline was selected and how the correction was applied.

Same comment for the section on spectral coherence.

There is a clear discrepancy in the description of these two methods and the detailed part on functional connectivity.

From the 15 animals two groups were constituted and compared: Well trained animals (n=8) and naïve animals (n=3), what about the 4 remaining animals? I do not understand the reason why the authors divide their group in two instead of using each rat as its own control. Moreover, the two groups are not comparable in size and the use of parametric statistics with n=3 is questionable (ANOVA for repeated measures).

Moreover, it is important to precise in the methods section how and when (which session) the naïve group and the well trained group were recorded.

Results:

The same lack of precision is also detrimental to results presentation. For example on figure 3, 4 and 5, what is represented in the graph is not precized (mean, SEM, STD).

Figure 2: Precise if the two spectrograms are normalized or not for each structure

Figure 3 & 4: The use of blue and red colors in these two figures really need to be rethought. It is very difficult to follow...and misleading.

What is the relation between this two figures ? The comparison of the odor response seems to me strange and I am just wondering if there is not an inversion in the results between the two figures and the two structures since activity of the PCx in the beta band is lower than the A1 whereas the graph is showing an inverse result...

How is the quantification performed on figure 4 ? On which period of time ?

Figure 7: To understand this figure it is really necessary to know more about how the selection of portion of signals where made in the disengaged situation.

Figure 8: How many trials for each animal ? This should be indicated close to each figure.

Figure 9: Same remark regarding the number of trials for each animal (n=4 only ) in each condition.

On this figure the significant clusters for OT9 seem to be quite different From the three others (negative value of the Z score, in a clear different frequency band: 8 Hz instead of between 1 and 2 Hz)

What does it mean ? This point should be described and discussed.

Discussion:

My major concern regarding this section is that it is disconnected from the results.

I would suggest that this section include a first paragraph with a synthesis of the experimental results and an attempt to relate the power analysis and the functional connectivity study.

Then the authors should stay in the line of their data to discuss more the results they obtained. For example, the fact that beta oscillations are context dependent in well trained animals but did not seem to be coherent between the two structures (even if nothing is said about PLI in this band).

Minor points:

Methods:

Line 88:“ The animals are referred to as OT1, OT2, etc...” On fig 8, the last animal is A10

Line 125: “in both the operant chamber...” A word is missing here

Reviewer 2:

This manuscript presents the results of an interesting set of observations in which rats that had previously acquired an auditory-olfactory associative go/no-go task showed significant multi unit, beta band oscillations in both the auditory cortex (A1) and the anterior olfactory cortex (aPCX). Functional coupling between A1 and aPCX was also demonstrated in the present study, with both evoked responses and functional connectivity between these two brain regions being expressed only in trained (as opposed to naïve) animals that were tested in the operant training chamber (as opposed to the home cage). The present study appears to have been carefully conducted, and the results have been appropriately analyzed and graphically portrayed in a clear manner. The manuscript is also very well written and includes an appropriate, conservative discussion of the relevant literature.

The results establish a behavioral model system for future studies of the mechanism whereby the observed linkage of the A1 and aPCX responses is established. As the authors point out in the Discussion (L299f), their new auditory-olfactory associative learning task in rats may be a useful model system in which to study the underlying mechanism whereby dementia disrupts the linkage between auditory signals (e.g., human language) and olfactory detection/discrimination capacity also demonstrated in dementing human subjects.

Minor changes: Mention when appropriate in Fig. captions that data are expressed as mean +/- SEM.

## Author Response

Dear Dr. Bakker,

We are excited that the reviewers and editors found our manuscript to be of interest for publication in eNeuro. We appreciate that Reviewer 1 recognized how our work “addresses for the first time the impact of the experimental context and subject engagement in the task on functional connectivity” and that Reviewer 2 found our study to be “carefully conducted”, the results “appropriately analyzed and graphically portrayed in a clear manner”, and the manuscript to be “very well written”. Encouraged by these comments, we have carefully considered the limitations of our submitted work, in order to strengthen the manuscript further. Below, we make a point-by-point response in blue font to each reviewer comment. The revised manuscript includes new text in yellow highlight. We hope that our efforts have made the manuscript acceptable for publication, and we are looking forward to your response.

Response to reviewer comments

Reviewer 1:

Methods:

This section is not precise enough and the part on signal processing need to be reorganized in a more rigorous way. For example, it is not clear how baseline LFP activity was determined both in the experimental cage and in the home cage. This point is critical as everything is then corrected by this baseline. Please provide a clear description of how baseline was selected and how the correction was applied. Same comment for the section on spectral coherence.

-We have clarified the method used for determining individual baselines (using periods of activity). Baseline adjustment procedure is now explained at three occasions on page 5. We hope that the reviewer feels that our methods are now presented with enough clarity.

There is a clear discrepancy in the description of these two methods and the detailed part on functional connectivity.

-The connectivity analysis currently occupies about 1 page of manuscript text, whereas the more commonly used electrophysiology methods, which will be more familiar to the readers of eNeuro, occupies about 1/2 page. Nonetheless, we have added additional detail where possible to help clarify differences between coherence analyses during trial-based activity and disengagement, and between homecage and operant chamber activity. We feel that the revised methods section is well balanced with regards to brevity, conciseness and need for methodological detail.

From the 15 animals two groups were constituted and compared: Well trained animals (n=8) and naïve animals (n=3), what about the 4 remaining animals? I do not understand the reason why the authors divide their group in two instead of using each rat as its own control.

-We have added additional information regarding the animals (pages 4-5); 4 animals were not able to perform the Go No-go task above criterion after electrode implants. Data from these animals were not considered further. In this revision we have doubled the number of naive animals to n=6 to be more comparable to our ‘expert’ group. As explained in the methods, unfortunately due to technical limitations were were unable to record from the same animals during the entire training regime wich lasted several weeks.

Moreover, the two groups are not comparable in size and the use of parametric statistics with n=3 is questionable (ANOVA for repeated measures).

Moreover, it is important to precise in the methods section how and when (which session) the naïve group and the well trained group were recorded.

-In the revised manuscript version, we have increased the number of Naive control animals to 6 (by adding 3 new animals). We believe that the increased sample size is sufficient for statistical comparison. We have clarified that Naive control recording data for analysis were taken from animals in their first 2-3 training sessions when they were still performing at chance levels (page 9 and Fig. 3 caption).

Results:

The same lack of precision is also detrimental to results presentation. For example on figure 3, 4 and 5, what is represented in the graph is not precized (mean, SEM, STD).

-We have clarified the results that are presented in Figures 3, 4, and 5, including SEM, etc.

Figure 2: Precise if the two spectrograms are normalized or not for each structure

-The spectrograms displayed in Fig. 2 show non-normalized raw data and are meant solely as representative examples of the raw data. Data used for analyses are processed as described elsewhere.

Figure 3 & 4: The use of blue and red colors in these two figures really need to be rethought. It is very difficult to follow...and misleading.

-We have changed the color scheme of Fig. 4 to grey and white, which we think helps to better communicate the pattern of results (pages 8-9).

What is the relation between this two figures ? The comparison of the odor response seems to me strange and I am just wondering if there is not an inversion in the results between the two figures and the two structures since activity of the PCx in the beta band is lower than the A1 whereas the graph is showing an inverse result...

We have now clarified that Fig. 3 displays the full FFT spectrum, while Fig. 4 shows the same data collapsed across the beta and gamma frequency bands for statistical analyses. The data have been updated since we doubled the n in this group. We do not see the inversion the reviewer notes, though now that we have changed the color scheme, the relationship will be more obvious. We have also changed the color scheme for Fig. 6 for the same reasons.

How is the quantification performed on figure 4 ? On which period of time ?

-The quantification was identical in Fig 3 and Fig 4; this is now clearly explained in the Fig 4 legend. We have now clarfied in the figure caption that activity was quantified 1) during the 500ms of tone presentation (tone-evoked activity, 2) for 250ms starting at tone offset and odor onset (odor evoked activity) and 3) during 500 ms of tone when the animals were in their home cage (passibve tone evoked activity).

Figure 7: To understand this figure it is really necessary to know more about how the selection of portion of signals where made in the disengaged situation.

-In the introduction, we operationalized the disengagement as periods where no task activity was present for at least 30 seconds. All animals displayed periods of task-activity intermixed with periods of disengagement from the task, periods where they were mostly grooming. We have explained this operationalization again on page 11.

Figure 8: How many trials for each animal ? This should be indicated close to each figure.

- In the original Fig 8, we plotted the PLI change instead of z score for Go and No-Go in the first two columns, using same number of trials for both conditions. Encouraged by the reviewer, we have now calculated the z score of the PLI change using all trials for each condition. When computing the direct comparison between Go and No-Go, we used the same number of trials for these two conditions. The content shown in figure 8 matches what is described in the methods and figure legend. We also added the number of trials for each rat and condition in Figures 8 and 9.

Figure 9: Same remark regarding the number of trials for each animal (n=4 only ) in each condition. On this figure the significant clusters for OT9 seem to be quite different From the three others (negative value of the Z score, in a clear different frequency band: 8 Hz instead of between 1 and 2 Hz). What does it mean ? This point should be described and discussed.

-We have noted on page 14 that OT9 shows a different pattern from the other animals. We do not know what caused this abberrant pattern for OT9.

Discussion:

My major concern regarding this section is that it is disconnected from the results.

I would suggest that this section include a first paragraph with a synthesis of the experimental results and an attempt to relate the power analysis and the functional connectivity study. Then the authors should stay in the line of their data to discuss more the results they obtained. For example, the fact that beta oscillations are context dependent in well trained animals but did not seem to be coherent between the two structures (even if nothing is said about PLI in this band).

-We have extensively modified the Discussion in the following ways:

1) paragraph 1 begins with a summary of our results and placement of these results in a broader context

2) We then discuss the LFP beta band power changes and speculate on mechanisms

3) We then discuss the functional connectivity changes and speculate on why the LFP changes and the functional connectivity changes occur in different frequency bands.

4) We end with further discussion of how these results related to broader, clinically relevant issues

Minor points:

Methods:

Line 88:“ The animals are referred to as OT1, OT2, etc...” On fig 8, the last animal is A10

-We have corrected this inconsistent labelling of individual animals, and thank the reviewer for pointing this out. We chose to label the animals OT (Odor-Tone) rather than A (for Animal) to avoid confusion with A1 (For primary auditory cortex).

Line 125: “in both the operant chamber...” A word is missing here

-We have completed this sentence “..both the operant chamber and in the home cage” (page 5).

Reviewer 2:

Minor changes: Mention when appropriate in Fig. captions that data are expressed as mean +/- SEM.

-Reviewer 2 asked us to clarify our Figures, similar to a point made by Reviewer 1. We have revised our figures and legends for increased clarity and comprehensibility, and we feel that the revised figures better communicate our results. We thank both reviewers for their helpful comments.
